# Ground-lying deadwood volume promotes soil beta diversity but not alpha diversity in European temperate forests

**DOI:** 10.1007/s11104-025-07600-6

**Published:** 2025-06-18

**Authors:** Mélody Rousseau, G. Arjen de Groot, Andrew K. Skidmore, Andjin Siegenthaler, Ivo Laros, Marco Heurich, Devara P. Adiningrat, Elnaz Neinavaz

**Affiliations:** 1 Faculty of Geo-Information Science and Earth Observation, University of Twente, 7522 NH Enschede, The Netherlands; 2 Wageningen Environmental Research, Wageningen University & Research, P.O. Box 47, 6700 AA Wageningen, The Netherlands; 3 Department of National Park Monitoring and Animal Management, Bavarian Forest National Park, Freyunger Str. 2, 94481 Grafenau, Germany; 4 Department of Forestry and Wildlife Management, Inland Norway University of Applied Sciences, Evenstads Vei 80, 2480 Koppang, Norway

**Keywords:** Coarse woody debris, Bark beetle, Bacteria, Fungi, Microfauna, Deadwood amount

## Abstract

**Background and aims:**

Deadwood plays a vital role in forest ecosystems, influencing soil biodiversity through nutrient enrichment and niche partitioning. While the effects of specific attributes of deadwood logs on soil biodiversity are well studied, it remains unclear whether and how the volume of deadwood affects soil biodiversity at the scale of forest stands. Additionally, the effects on soil biodiversity may differ between gradually accumulated deadwood and large volumes resulting from sudden stand-level disturbance events. In this study, we aim to assess such effects on alpha and beta diversity of soil microbes and microfauna.

**Methods:**

Soil samples were gathered from forest plots following a gradient of deadwood volumes in European temperate forests in Germany and The Netherlands. Using extracellular DNA metabarcoding, we analysed the soil diversity of bacteria, fungi and microarthropods. For the Bavarian Forest National Park, we also compared the diversity patterns of these biotas between areas affected by bark beetle outbreaks and unaffected areas of Norway spruce forest.

**Results:**

Increased deadwood stock had a marginal effect on soil microbial and microarthropod community composition, with no impact on overall diversity. Bark beetle-affected areas had distinct soil communities, with a lower fungal and microarthropod diversity.

**Conclusion:**

Our study provides the first insights into soil diversity patterns associated with increased deadwood volume at the forest stand. While shifts in soil biodiversity composition were minimal, the retention of deadwood in European temperate forests can promote heterogeneity in soil communities. Furthermore, changes in soil biodiversity following bark beetle outbreaks may have long-term consequences on forest regeneration.

**Supplementary Information:**

The online version contains supplementary material available at 10.1007/s11104-025-07600-6.

## Introduction

Deadwood stock depends on several factors, such as stand age (Bujoczek et al. 2021), climate and elevation (Castagneri et al. 2010), and forest management practices (Lombardi et al. 2008). Given its many benefits for forest biodiversity, increasing deadwood stock has become a key objective in managing European temperate forests (Bujoczek et al. 2021). Deadwood is crucial in forest ecosystems by supporting biodiversity across a range of organisms, including lichens, bryophytes, fungi, birds, and mammals (Heilmann-Clausen and Christensen 2005; Stokland et al. 2012). It positively influences forest biodiversity by increasing landscape heterogeneity, thereby providing suitable habitats for a higher diversity of insects and deadwood-dependent species (Heilmann-Clausen and Christensen 2005; Lassauce et al. 2011; Sacher et al. 2022). In addition, a higher plant diversity emerges with increasing deadwood, as its decomposition and mineralisation enhance soil nutrient levels (Winter et al. 2015).

The decomposition and mineralisation of deadwood impact soil microbial and microarthropod communities, as well as ecosystem functions (Bani et al. 2018; Seastedt 1984). Saprotrophic fungi, for example, decompose deadwood by breaking down complex organic materials (i.e., lignin, cellulose and hemicellulose) into simpler compounds, contributing to soil formation, nutrient cycling and habitat creation for other organisms (Baldrian et al. 2016). Bacteria further enhance these processes by acting as decomposers, nutrient recyclers, and fungal symbiotic partners, facilitating efficient breakdown of deadwood and cycling of nutrients within and between ecosystems (Bani et al. 2018). The microbial communities serve as essential food sources for the soil fauna. For example, microarthropods predominantly feed on fungal mycelia, either directly by grazing on it or indirectly through consuming and mineralising dead organic matter (Boddy and Jones 2008). As a result, microphytageous microfauna has been found in higher densities near logs (Čuchta et al. 2019; Huhta et al. 2012). The positive effects of deadwood on soil biodiversity have primarily been observed in soil communities living directly beneath or near logs, where its influence is the strongest (Huhta et al. 2012; van der Wal et al. 2017; Wang et al. 2023). However, studies examining the effects of deadwood at greater distances (> 8 m from log; Minnich et al. 2021) are limited. It remains unclear, therefore, whether deadwood continues to affect soil biodiversity at the broader forest stand level, where its effects are less direct.

At the stand level, the deadwood amount is driven by various processes. Deadwood accumulates slowly due to small-scale events (e.g., natural senescence, competition, drought, grazing pressure) (Bobiec 2002; Mountford et al. 1999), resulting in a low-level, semi-random distribution of deadwood (Rousseau et al. 2024a). In contrast, large-scale disturbance, such as windthrow or dieback caused by the European spruce bark beetle (*Ips typographus L.*) (Custer et al. 2020), can lead to large and sudden amounts of deadwood, which may impact soil biodiversity differently than gradually accumulated deadwood. Disturbed areas (e.g., by bark beetle) undergo significant changes in their forest and community structure, including increased light availability due to the opening of the canopy, high tree mortality resulting in large amounts of deadwood, and greater coverage of herbaceous and shrub plant species (Winter et al. 2015). These changes lead to significant alterations in soil conditions (Štursová et al. 2014), and influence soil biodiversity (Ghandi et al. 2022). However, the specific effects on soil microbial and microarthropod communities remain unclear and site-dependant. Studies on bacterial, fungal and microarthropod communities show inconsistent effects of bark beetle-induced mortality on soil biodiversity, with some studies showing positive effects (Custer et al. 2020; Mayer et al. 2022; Mikkelson et al. 2016), while others show neutral to negative effects (Beudert et al. 2014; Ferrenberg et al. 2014; Kosunen et al. 2020; Pec et al. 2017; Winter et al 2015). With large-scale bark beetle outbreaks occurring more frequently – potentially exacerbated by climate change – it is increasingly important to understand how both gradual and sudden accumulation of deadwood influence soil biodiversity for effective forest management (Hlásny et al. 2021).

In this study, we investigate the responses of topsoil bacteria, fungi and microarthropods (mites and springtails) to variations in deadwood volume at the forest stand level (30 × 30 m). Soil biodiversity, especially soil microbial communities, is strongly influenced by forest type and soil properties (Lladó et al 2018), which can, in turn, modulate the impact of deadwood on the soil biodiversity. Therefore, we included these factors in our analysis, as they may influence how deadwood affects soil biodiversity through nutrient enrichment, microbial diversity, and biodiversity specialisation (Błońska et al. 2019; Lladó et al 2018; Rousk et al. 2010; Rousseau et al. 2024b). Using metabarcoding, we assess changes in soil taxonomic diversity along a gradient of ground-lying deadwood in European mixed temperate forests in Germany and The Netherlands (objective 1). Additionally, we focus on the Bavarian Forest National Park (Germany), an area impacted by bark beetle outbreaks, comparing soil taxonomic diversity in dieback Norway spruce stands to that in undisturbed stands to characterise soil biodiversity shifts following bark beetle disturbances (objective 2). We hypothesise that: i) soil biodiversity will increase with deadwood volume, with certain bioindicators associated with forest stands that contain high deadwood levels; and ii) bark beetle-affected areas will support higher soil biodiversity and exhibit distinct communities compared to undisturbed areas, with some biotic groups, such as fungi, showing stronger responses than others.

## Materials and methods

### Study site description

The Bavarian Forest National Park (here referred to as BP; 48.9597° N, 13.3949° E) and the Neuburg Forest (here referred to as NF; 48.3310° N, 13.204° E) are both located in southeastern Germany along the Czech Republic border. The Bavarian Forest Nation Park (BP) is a national park that encompasses an area of approximately 250 km^2^, while the Neuburg Forest (NF) covers a smaller area of about 186 km^2^. Both forested areas are shaped by a combination of continental and temperate climates (Heurich et al. 2010). The underlying parent material consists of gneiss and granite, developing into oligotrophic and acidic soils (van der Knaap et al. 2019). These soils primarily include spodo-dystric cambisol and dystric histosol soils (Panagos et al. 2012). Ranging in elevation from 600 to 1,450 m, the Bavarian Forest Nation Park is dominated by European beech (*Fagus sylvatica*) and Norway spruce (*Picea abies*) at higher altitudes and silver fir (*Abies alba*) at low and intermediate altitudes (Cailleret et al. 2014). The Bavarian Forest Nation Park has embraced a nature-oriented management approach, i.e., a strict non-intervention strategy in the park’s designated natural zone (75% of the area). The remaining part of the park is designed as a management zone where conservation measures can be actively implemented (van der Knaap et al. 2019). For instance, active logging for economic purposes was discontinued in old-growth and mature stands. However, limited-scale preventive logging for bark beetle management has been allowed in the management zone adjacent to commercially managed forests in the vicinity (Müller and Bütler 2010). The Neuburg Forest is a semi-production forest with some protected areas for recreational use and conservation. Active logging is conducted throughout the forest. With an elevation between 400 and 500 m, it is mainly composed of European beech (*Fagus sylvatica*), Norway spruce (*Picea abies*), and English oak (*Qercus robus)*. The dominant soil types are similar to the ones in the Bavarian Forest Nation Park (spodo-dystric cambisol and dystric histosol soil).

The Hoge Veluwe National Park (here referred to as HV; 52.0787° N, 5.8325° E), the Veluwezoom National Park (here referred to as VZ; 52.0483° N, 6.0199° E) and Royal Estate Het Loo (here referred to as KD; 52.1400° N, 5.5600° E) are two national parks, and one domain forest located in the central part of The Netherlands. They comprise a combination of forest, heathland, drift sand, and grassland ecosystems, encompassing about 140 km^2^ ha of forest (Hein 2011). The area is characterised by a temperate maritime climate and an elevation of approximately 40 m, featuring minimal topographic variation. The dominant vegetation includes Scots pine (*Pinus sylvestris*), Norway spruce (*Picea abies*), English oak (*Quercus robur*), silver birch (*Betula pendula*), and European beech (*Fagus sylvatica*) growing on oligotrophic, sandy and acidic soils with a low buffering capacity. The prevalent soil types consist of ferro-humic and orthic podzols, as well as calcaric regosol soils (Panagos et al. 2012). The management strategies implemented in the Veluwe region aim to support and sustain a balance between the historical-cultural, recreational, and biodiversity values, though differ between parks (Rousseau et al. 2024a). One approach to promoting biodiversity is increasing deadwood stock. More specifically, deadwood removal is permitted to attain a more natural and diverse forest composition and structure, preserve traditionally more open woodland landscapes, enhance landscape aesthetics, improve recreational opportunities, eradicate exotic trees, and support small-scale sustainable logging landscapes (Het Nationaal Park De Hoge Veluwe 2015; Kroondomein Het Loo 2022; Nationaal Park Veluwezoom en IJsseluiterwaarden 2014).

### Data collection

**Fig. 1:**
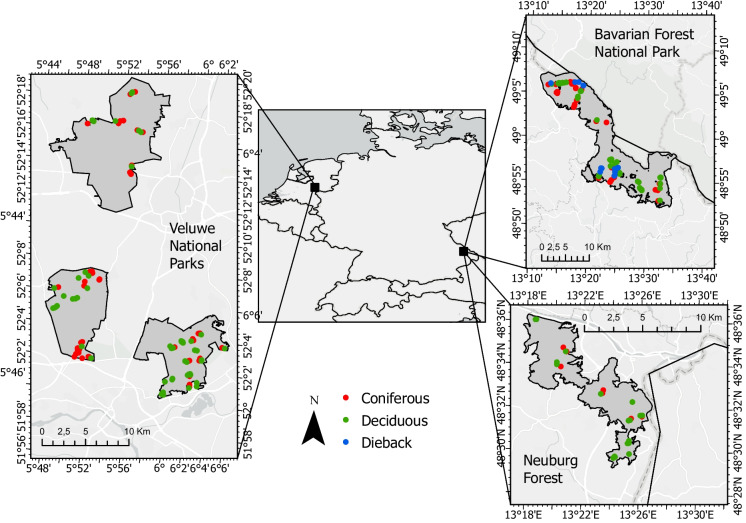
Maps of the study sites show plot locations in Germany and The Netherlands. Sources: Esri, TomTom, Garmin, FAO, NOAA, USGS, © OpenStreetMap contributors, and the GIS User community

A total of 191 soil samples were collected during the summers of 2020, and 2021 in Germany and The Netherlands (Fig. 1). Sampling plots (30 m x 30 m) were stratified over forest-type dominance, defined as more than 75% dominance of either coniferous or deciduous trees. The sampling strategy aimed to capture a gradient of deadwood volume randomly distributed over the landscape, modified by purposive sampling where access was poor (Fig. S1 and S2). In 2021, this resulted in 87 coniferous plots and 89 deciduous plots sampled (objective 1). Additionally, in 2020, 15 coniferous plots and 15 Norway spruce dieback plots were sampled along an elevational gradient in BP (objective 2). These dieback plots represented areas affected by bark beetle outbreaks over the past 3 to 25 years (Latifi et al. 2021) (see Fig. S3).

For each plot, two 3 m × 3 m subplots were randomly established, independently of the ground-lying deadwood distribution in the plot. From each subplot, one composite sample was collected from the mineral horizon (after removal of litter), consisting of nine topsoil cores with a diameter of 5 cm and a depth of 10 cm, following a systematic sampling design. Samples were kept on ice in the field and frozen at −20 °C until further processing. The soil corer was bleach-sterilized (10% bleach) and rinsed with deionised water between samples to prevent cross-contamination. Field controls were collected every fifth plot and consisted of an aliquot of the deionised water rinse.

Soil pH (H_2_O) was determined with a pH meter (Metrohm 914 pH and conductivity meter) in accordance with the ISRIC protocol (Black 1965; van Reeuwijk 2002). Total nitrogen (TN), total carbon (TC), C:N ratio and soil organic matter content (SOM) were also determined following Rousseau et al. (2024b). For the 2021 plots, ground-lying deadwood volume was estimated using line-intersect sampling (LIS) (Rousseau et al. 2024a). The LIS inventory was conducted within each plot using two fixed transect lines of 42.4 m perpendicular to each other in northeast-southwest and northwest-southeast directions. Only ground-lying deadwood logs intersected by a fixed transect line and with a minimum diameter *d* of 10 cm (IPCC 2003; Rondeux et al. 2012) were recorded. For each log, the diameter intersecting with a transect line was measured. The total deadwood volume was calculated using de Vries’formula (de Vries 1986; Van Wagner 1968):

$$V={\pi }^{2}\sum \left(\frac{{d}^{2}}{8L}\right)*\text{10,000}$$

where V represents the area-based volume of the plot (m^3^ ha⁻^1^), *d* is the diameter of the deadwood log at the point of intersection with the transect line (m), and L is the total length of the transects within the plot (two transect lines of 42.4 m each). Ground-lying deadwood volume was not determined for the 2020 plots since these plots were only used to determine the impact of bark beetle dieback.

### DNA extraction and amplification

The saturated phosphate buffer method (Taberlet et al. 2012) was used for the extracellular DNA extraction of soil samples. To 15 g of soil, 15 mL of freshly prepared saturated phosphate buffer solution (Na_2_HPO_4_; 0.12 M; pH ≈ 8) was added and thoroughly mixed for 10 min. Following a centrifugation step, the supernatant was further processed with NucleoSpin® Soil kit (Macherey–Nagel, Düren, Germany) per the manufacturer’s protocol instructions. The saturated phosphate buffer replaced the kit’s lysis steps. An additional purification step was introduced while processing the 2021 samples to minimise PCR dropout (i.e., failed amplification) experienced with the 2020 samples. This was especially noticeable with microarthropod DNA, where approximately 25% of samples failed to amplify. For every 22 extractions, a negative extraction control, defined as the saturated phosphate buffer solution, was included.

The DNA concentration of each sample was quantified with the Synergy™ HTX multi-mode microplate reader, and DNA extracts were further standardised to 5 ng/µl for all three taxonomical groups as well as diluted 100 × for bacteria and fungi only. Field and negative controls were also included and pooled for each sampled region. The primer sets 515F/806R (Apprill et al. 2015; Parada et al. 2016), ITS86/ITS4-ngs (Tedersoo et al. 2014), and MiteMinBarF7/MiteMinBarR4 specifically targeting mites and springtails (de Groot et al. 2016) were used to amplify the V4 region of the 16S rRNA gene of bacteria, the 5.8S and ITS2 rRNA region of fungi and the subunit 1 of the COI rRNA region of microarthropods, respectively. CS1/CS2 adaptor sequences (Fluidigm Access Array System, Fluidigm, South San Francisco, CA) for multiplexing were included in the primers. A detailed description of the amplification process can be found in Table S1. Amplicons were sent to Genome Quebec (Montreal, Canada) for multiplexing (using i5/i7), purification of the PCR products (sparQ magnetic beads), library preparation, and high throughput sequencing using the Illumina NovaSeq 6000 SP platform (PE250 kit).

A sequence error was identified in the primer set (old primer: CS1_MiteMinBarF7: ACACTGACGACATGGTTCTACACAT**CG**ITTYRTIATRATTTTTTTYATAG) used for COI rRNA region amplification of 2020 samples. This error was corrected in an adjusted primer set (adjusted primer: CS1_MiteMinBarF7: ACACTGACGACATGGTTCTACACAT**GC**ITTYRTIATRATTTTTTTYATAG), which was used for sequencing samples collected during the 2021 campaign. A subset of 30 samples was subjected to comparative analysis using both the old and adjusted COI primer sets. Wilcoxon signed-ranked tests showed no significant differences in OTU richness (N = 60, W = 483, *p-value* = 0.631; Fig. S4.A) and diversity (N = 60, W = 547, *p-value* = 0.155; Fig. S4.B). Although PERMANOVA analysis highlighted minor but significant differences in community structure (R^2^ = 0.02, F = 1.286, *p-value* = 0.001), the computed NMDS showed that, except for three samples, all other pairs of samples cluster together (Fig. S4.C). Based on these results, we consider the COI 2020 data to be reliable.

### Data processing and statistical analysis

All statistical analyses were performed using R version 4.4.1 (https://www.R-project.org/). All graphics were generated using the *ggplot2* v.3.4.2 package (Wickham 2016), and all statistical analyses were conducted on the rarefied samples unless specified otherwise. The bioinformatics analyses of the raw sequence data were conducted using the QIIME 2™ software (Bolyen et al. 2019) as described in Rousseau et al. (2024b). The SILVA database (Quast et al. 2013), the UNITE database (Nilsson et al. 2019), and the BOLD database (Ratnasingham and Hebert 2007) were used for taxonomical assignments of bacterial ASVs (Amplicon Sequence Variants), fungal ASVs and microarthropod OTUs (Operational Taxonomical Units), respectively, followed by LULU post-clustering curation (Frøslev et al. 2017). The BOLDigger package was used for the taxonomical assignment of microarthropod OTUs with a 98% similarity (Buchner and Leese 2020). More detailed information about the bioinformatic pipeline parameters and results can be found in Table S2. Prior to data analysis, only COI reads belonging to mites (Acarii), and springtails (Collembola) OTUs were filtered and retained. Finally, curated ASV and OTU tables were rarefied to 82,825 reads (bacteria), 77,469 reads (fungi), and 5,197 reads (microarthropod) per sample (based on minimum read counts), averaging 100 rarefactions performed with the *rrarefy* function (package *vegan* v.2.6–4; Oksanen et al. 2008). Alpha-diversity metrics (observed number of ASVs/OTUs and Shannon index) for bacteria, fungi and microarthropod were calculated with the *estimate_richness* function from the *phyloseq* v.1.44.0 package (McMurdie and Holmes 2013). Prior to data analysis, variables measured at the subplot level (i.e., read counts of curated ASV/OTU tables and soil properties) were averaged at the plot level. All analyses were conducted separately for all three taxonomical groups.

We first examined how a gradient of deadwood volume influenced soil taxonomic biodiversity (objective 1) in coniferous and deciduous plots sampled during the 2021 field campaign. Pearson correlations between deadwood volume and soil properties (pH, SOM, TC, TN, C:N ratio) were conducted to assess the relation between deadwood volume and soil biochemistry. Soil properties significantly correlated with deadwood volume were independently tested for their influence on soil biodiversity, to determine the indirect effect of deadwood volume, using multiple linear regression models for alpha-diversity metrics and PERMANOVA analyses for community composition separately for each taxonomical group. Multiple linear regression models were performed on alpha-diversity metrics with deadwood volume and deadwood volume x forest type (coniferous vs. deciduous) as fixed effects. Soil properties not correlated with deadwood volume, park identity (park) and forest type were also included as covariates. Continuous variables (deadwood volume and soil pH) were scaled, and alpha-diversity metrics were log-transformed to meet model assumptions. PERMANOVA analyses (Bray–Curtis dissimilarity of Hellinger-transformed read counts; 999 permutations) were performed to assess changes in community composition at the ASV/OTU level using the same variables using the *adonis2* function from the package *vegan* v.2.6–4. To identify bioindicators of deadwood volume, differential abundance analysis was performed at both the ASV/OTU and family levels using the *ALDEx2* package (centre log-ratio of raw read counts; Fernandes et al. 2014). The *aldex.corr* function was used, and only ASVs/OTUs and families with a *p-value* < 0.05 (Benjamini–Hochberg correction) were considered discriminant.

Next, we compared soil biodiversity between undisturbed Norway spruce and disturbed stands (dieback Norway spruce) sampled during the 2020 field campaign in the Bavarian Forest National Park (BP) (research question 2). Since normality and homogeneity of variances were not met, Wilcoxon rank-sum tests were used to compare alpha-diversity metrics. Differences in community composition were assessed with PERMANOVA analysis (Bray–Curtis dissimilarity of Hellinger-transformed read counts; 999 permutations) at the ASV/OTU level. NMDS (Non-Metric Multidimensional Scaling) was used to visually represent the differences between undisturbed and disturbed stands. Differential abundance analysis was also conducted at the family level using the *aldex.ttest* function from the *ALDEx2* package to identify bioindicators of disturbed stands. Disturbed stands were in areas impacted by bark beetle outbreaks over the last 3 to 25 years (Latifi et al 2021). The effect of time since infestations on alpha-diversity metrics and community composition were tested with simple linear regression models and PERMANOVA analysis (Bray–Curtis dissimilarity of Hellinger-transformed read counts; 999 permutations) at the ASV/OTU level. Differences in soil chemistry (soil pH, SOM, TC, TN and C:N ratio) were also compared between disturbed and undisturbed areas using unpaired t-tests.

## Results

### Alpha-diversity and community composition across parks

Variations in bacterial, fungal and microarthropod alpha-diversity metrics (richness and diversity) were observed between the five parks, with the Bavarian Forest National Park (BP) showing the highest richness and diversity across all taxonomical groups (Table 1). The community composition of the three taxonomical groups was characterized by the same dominant groups across parks, with the highest similarity between parks within the same country (Fig. S5). However, the relative abundance of dominant bacterial, fungal, and microarthropod families varied greatly among parks. For example, the bacterial family Acidobacteriaceae GP1 ranged from 3.69% (BP) to 14.49% (HV), the fungal family Russulaceae from 7.74% (HV) to 38.47% (NF), and the microarthropod family Entomobryidae from 0.16% (VZ) to 26.00% (BP).

**Table 1:** Number of sampled plots (N), mean ± sd (range) of deadwood volume (m^3^ ha^−1^), mean ± sd for each soil property (pH, organic matter content, total carbon, total nitrogen, and C:N ratio), observed richness (mean ± sd) and Shannon diversity index (mean ± sd) for all three taxonomical groups per park. NF: Neuburg Forest. BP: Bavarian Forest National Park. HV: Hoge Veluwe National Park. KD: Royal Estate Het Loo. VZ: Veluwezoom National Park

	NF 2021	BP 2021	HV 2021	KD 2021	VZ 2021	BP 2020
N	26	38	37	18	42	30
deadwood volume	23.40 ± 40.09 (0.00–187.85)	52.42 ± 61.63 (1.57–299.23)	23.49 ± 19.19 (0.00–95.59)	12.47 ± 12.45 (0.00–35.23)	26.94 ± 23.42 (0.00–84.30)	NA
soil pH	3.85 ± 0.21	3.55 ± 0.24	3.25 ± 0.20	3.25 ± 0.11	3.28 ± 0.20	3.51 ± 0.30
SOM	4.59 ± 0.85	14.51 ± 8.90	13.36 ± 6.81	8.67 ± 4.64	11.88 ± 6.31	23.08 ± 11.82
TN	0.25 ± 0.19	0.62 ± 0.41	0.52 ± 0.26	0.37 ± 0.21	0.51 ± 0.23	1.13 ± 0.56
TC	3.65 ± 2.65	13.03 ± 9.67	13.79 ± 7.37	8.76 ± 4.50	12.04 ± 6.21	24.03 ± 13.97
C:N ratio	16.57 ± 3.83	20.40 ± 2.72	26.47 ± 4.06	26.09 ± 5.19	23.02 ± 3.10	20.68 ± 3.21
16S – Richness	18,102 (2,306 ± 561)	26,788 (2,754 ± 540)	17,223 (1,959 ± 635)	11,156 (1,962 ± 430)	19,988 (2,073 ± 607)	31,596 (3,630 ± 762)
16S – Diversity	6.47 ± 0.45	6.70 ± 0.32	6.21 ± 0.45	6.22 ± 0.38	6.26 ± 0.46	7.00 ± 0.35
ITS – Richness	1,018 (81 ± 25)	1,363 (96 ± 39)	2,213 (160 ± 49)	1,075 (128 ± 58)	1,938 (135 ± 48)	2,133 (164 ± 47)
ITS – Diversity	2.85 ± 0.50	3.01 ± 0.53	3.52 ± 0.45	3.39 ± 0.63	3.35 ± 0.56	3.60 ± 0.37
COI – Richness	336 (46 ± 20)	503 (61 ± 18)	469 (71 ± 19)	308 (66 ± 26)	446 (63 ± 24)	562 (85 ± 30)
COI – Diversity	2.00 ± 0.71	2.38 ± 0.48	2.38 ± 0.40	2.44 ± 0.39	2.42 ± 0.47	2.84 ± 0.50

### Soil biodiversity response to deadwood volume variation

Soil biodiversity composition and alpha-diversity metrics of the 2021 sample campaign across all five parks were poorly associated with gradients of deadwood volume. For all taxonomical groups, multiple linear regression models showed no significant relationship between deadwood volume and alpha-diversity metrics (Table 2). Even though the interaction effect between deadwood volume x forest type was significantly related to fungal richness, post-hoc tests per forest type did not show any significant effects of deadwood volume (coniferous: *p* = 0.085; deciduous: *p* = 0.052; Table S3). Bacterial and fungal diversity were the most strongly influenced by park identity, forest type, soil pH and C:N ratio, while only forest type was associated with significant variations in microarthropod diversity (Table 2).

**Table 2:** Results of multiple linear regression models for soil pH, C:N ratio, park identity (BP, HV, KD, VZ compared to NF), deadwood volume (m^3^ ha^−1^), forest type (coniferous vs. deciduous), and the interaction deadwood volume x forest type on the observed richness and Shannon diversity index of all three taxonomical groups. Scaled regression estimates (β) are indicated for each fixed effect. Bold regression estimates and Adjusted R^2^ refer to significant p-values (* < 0.05; ** < 0.01; *** < 0.001). NS denotes non-significant fixed effects. NF: Neuburg Forest. BP: Bavarian Forest National Park. HV: Hoge Veluwe National Park. KD: Royal Estate Het Loo. VZ: Veluwezoom National Park

	Observed richness			Shannon index		
	*16S*	*ITS*	*COI*	*16S*	*ITS*	*COI*
Adjusted R^2^	0.465***	0.325***	0.235***	0.332***	0.202***	0.058*
Intercept	7.353***	4.272***	3.89***	1.787***	1.018***	0.674***
BP	0.391***	0.217*	0.225*	0.084***	0.078	0.174*
HV	0.346***	0.961***	0.482**	0.0742**	0.344***	NS
KD	0.351***	0.733***	0.324*	0.069*	0.318***	NS
VZ	0.291***	0.810***	0.330*	0.012*	0.283***	NS
soil pH	0.149***	0.150**	NS	0.033***	0.050*	NS
C:N ratio	−0.110***	NS	NS	−0.023**	NS	NS
forest type	NS	−0.278***	-0.172*	NS	−0.135***	NS
deadwood volume	NS	NS	NS	NS	NS	NS
deadwood volume x forest type	NS	0.161*	NS	NS	NS	NS

**Fig. 2:**
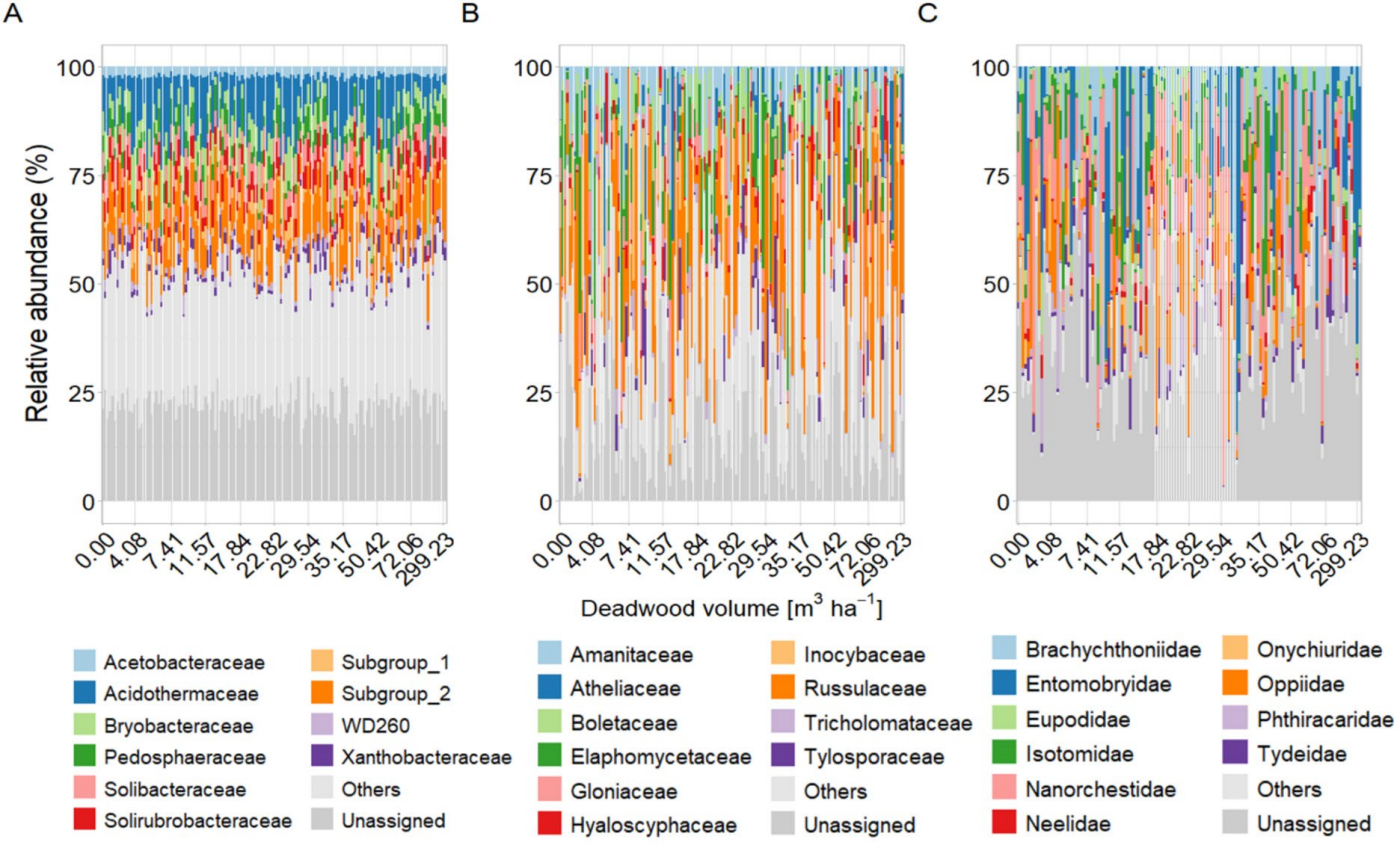
Relative abundance (%) of the top ten bacterial (**A**), fungal (**B**) and microarthropod (**C**) families along the increasing deadwood volume (m^3^ ha^−1^) for each sample

Deadwood volume was significantly correlated with bacterial, fungal and microarthropod community composition, as well as fungal communities in both coniferous and deciduous stands (Tables 3 and S4). However, deadwood volume only explained 1 to 3% of the variation in community composition. When testing bioindicators of deadwood volume, the differential abundance analysis conducted at the ASV (bacteria and fungi) and OTU levels (microarthropod), as well as family levels, did not produce significant bioindicators. This implies that no specific taxa were conclusively linked to deadwood volume. This is consistent with the absence of patterns in relative abundances of abundant families along the deadwood volume gradient (Fig. 2). Contrary to deadwood volume, park identity and soil pH accounted for a larger part of the variation in community composition, explaining between 10–18% and 5–15%, respectively.

**Table 3:** Results of PERMANOVA (Bray–Curtis dissimilarities constructed from Hellinger-transformed reads; 999 permutations) analysis for soil pH, C:N ratio, park identity (park), deadwood volume (m^3^ ha^−1^), forest type (coniferous vs. deciduous), and the interaction deadwood volume x forest type on the community composition of all three taxonomical groups. Bold R^2^ refer to significant p-values (* < 0.05; ** < 0.01; *** < 0.001). NS denotes non-significant fixed effects

	park	soil pH	C:N ratio	forest type	deadwood volume	deadwood volume x forest type
16S	0.16***	0.15***	0.04***	0.04***	0.02***	NS
ITS	0.10***	0.05***	0.02***	0.05***	0.01**	0.01*
COI	0.18***	0.13***	0.03***	0.04***	0.03***	NS

Deadwood volume was weakly but positively correlated with SOM (cor = 0.19, *p* = 0.016), TN (cor = 0.22, *p* = 0.006), and TC (cor = 0.16, *p* = 0.048) but not with C:N ratio (cor = −0.06, *p* = 0.421) or soil pH (cor = 0.024, *p* = 0.760). Bacterial, fungal and microarthropod alpha-diversity metrics were significantly influenced by TC and TN, but not SOM or soil pH (Table S5). Additionally, SOM, TC, and TN were significantly correlated with bacterial, fungal, and microarthropod community composition, explaining between 2 and 6% of the observed variation (Table S6).

### Distinct soil biodiversity following bark-beetle outbreaks in the Bavarian Forest National Park

**Fig. 3:**
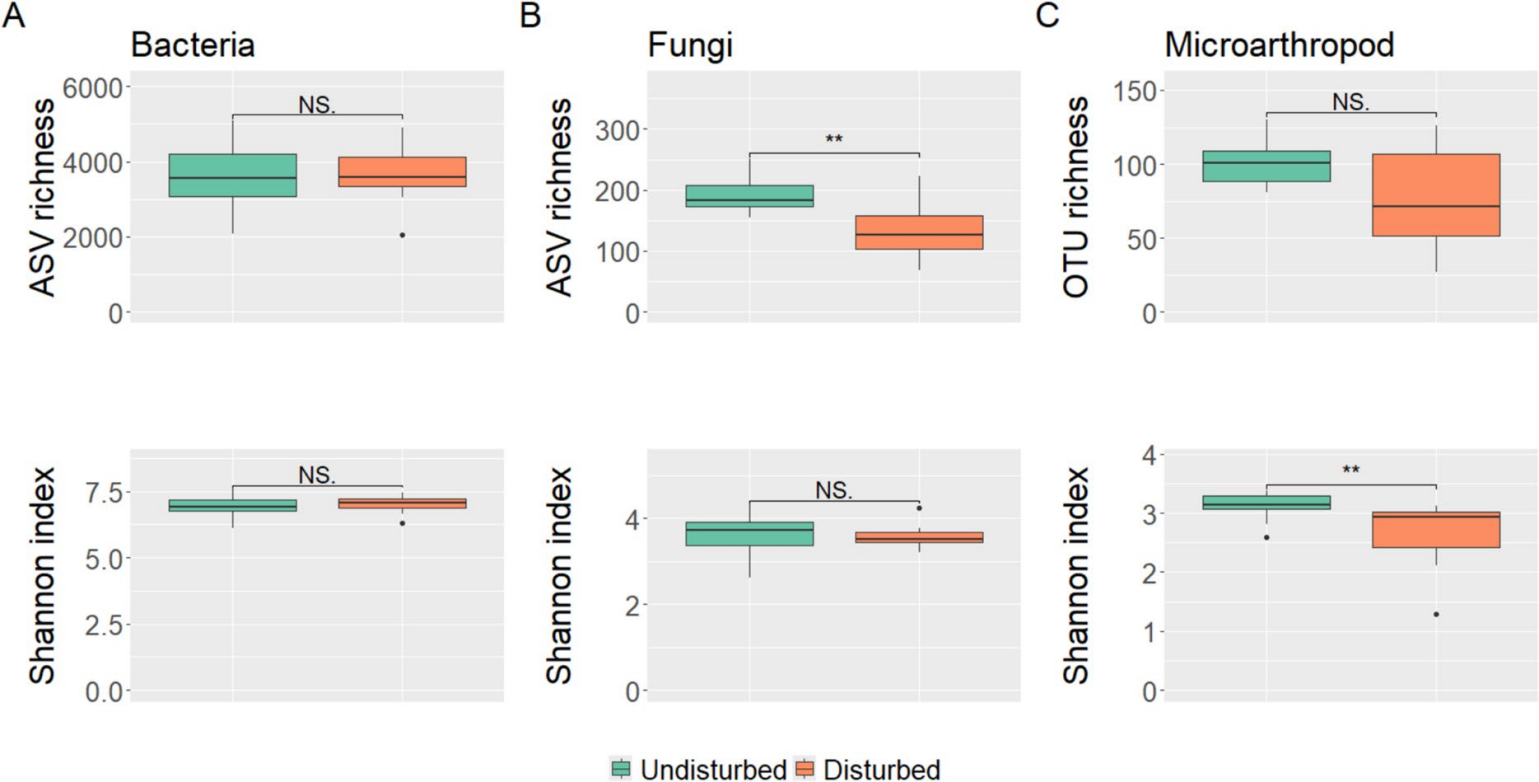
Differences in observed richness and Shannon diversity index for bacteria (**A**), fungi (**B**), and microarthropods (**C**) between undisturbed and disturbed stands in the Bavarian Forest National Park. Results of Wilcoxon rank-sum tests are indicated with significant *p-values* as follows: * < 0.05; ** < 0.01; *** < 0.001. NS denotes non-significant differences

**Fig. 4:**
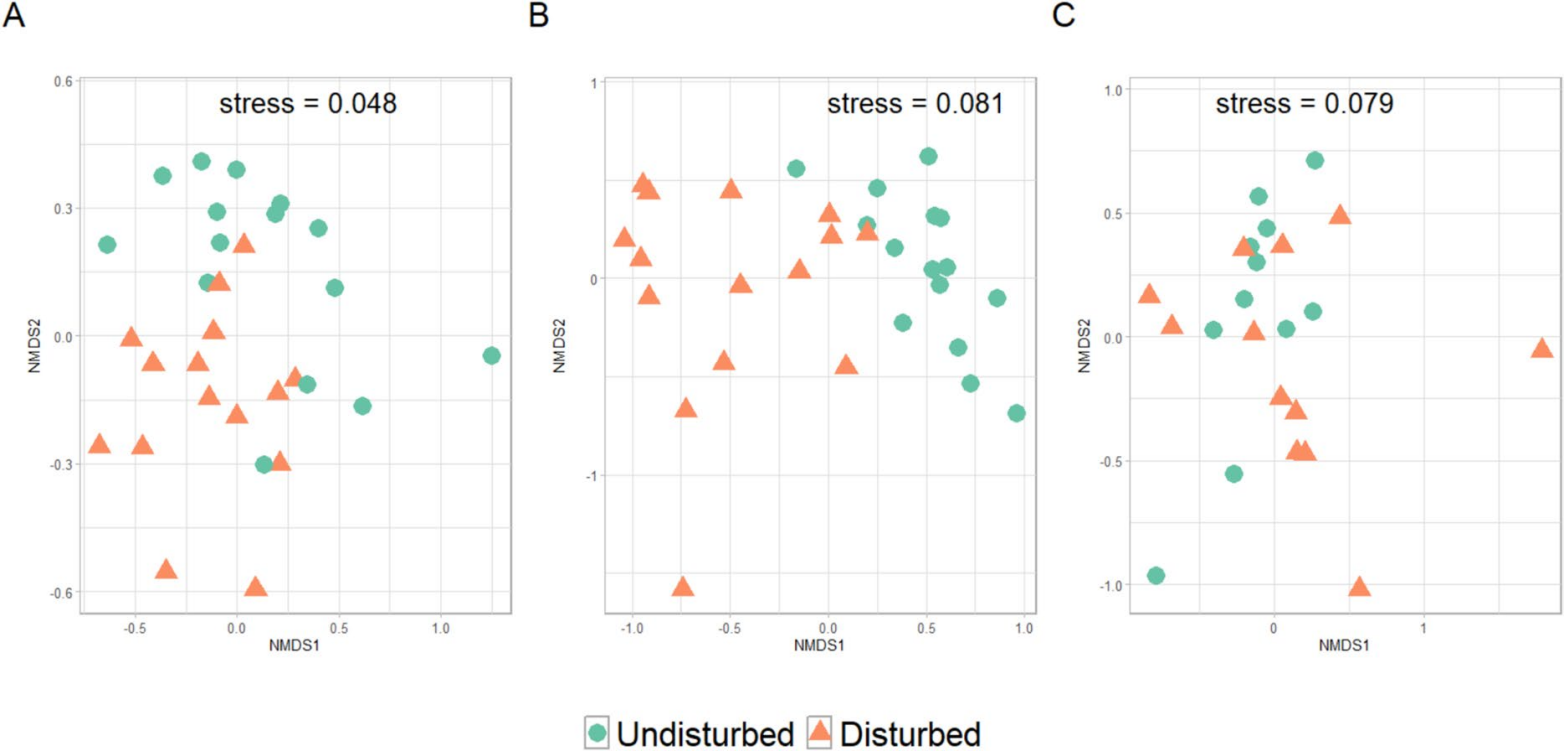
NMDS plot based on the Bray–Curtis dissimilarity (Hellinger-transformed reads) of bacterial (**A**) and fungal (**B**) communities at the ASV level and microarthropod communities (**C**) at the OTU level in the Bavarian Forest National Park between undisturbed and disturbed stands

**Fig. 5:**
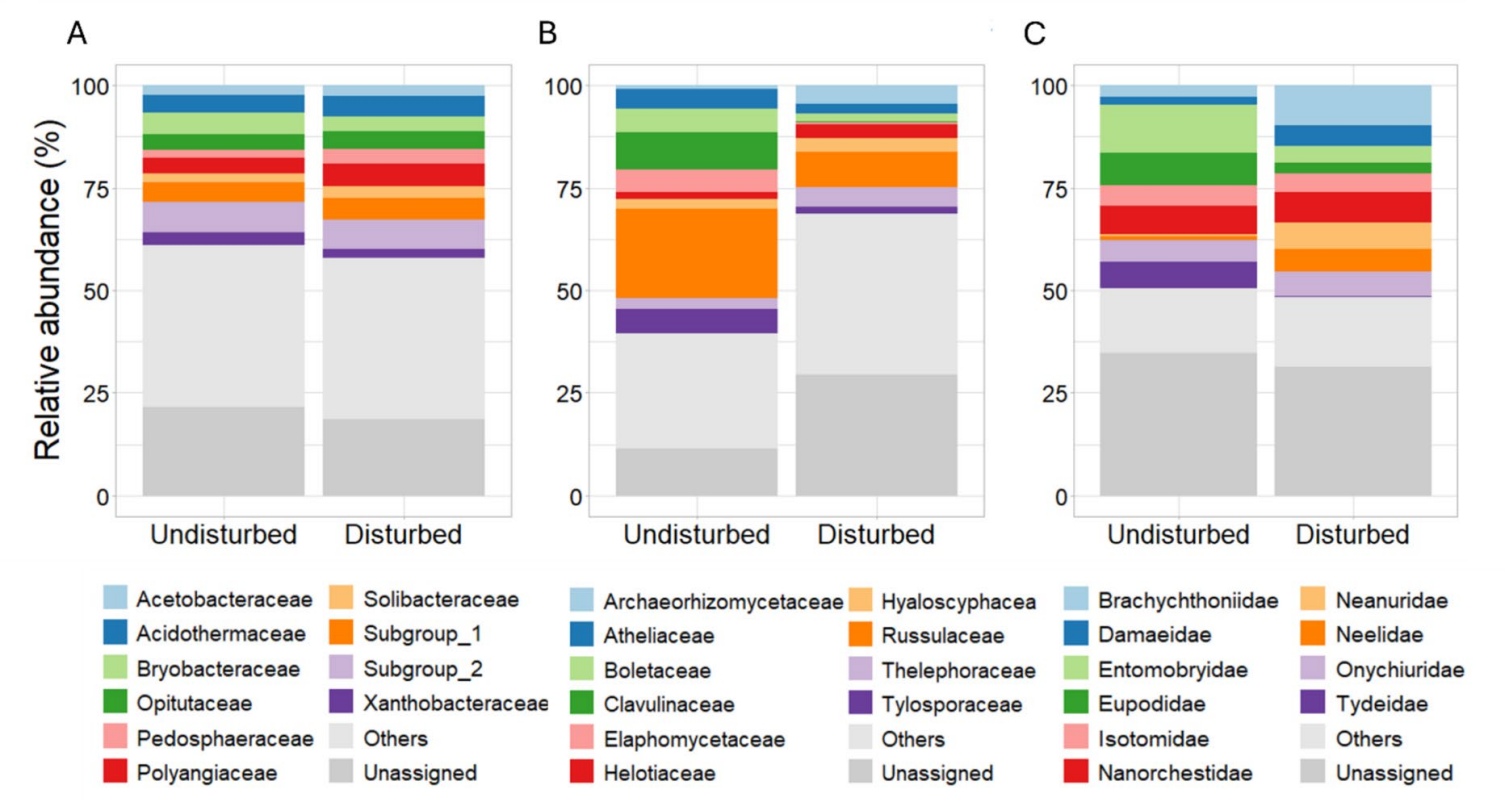
Mean relative abundance (%) of the top ten families for bacteria (**A**), fungi (**B**) and microarthropod (**C**) between undisturbed and disturbed stands at the family level in the Bavarian Forest National Park

Disturbed stands resulting from bark beetle outbreaks exhibited distinct soil biodiversity compared to undisturbed stands in the 2020 dataset. Soil biodiversity richness and Shannon diversity index were marginally influenced by the bark beetle outbreaks. Only fungal richness and microarthropod diversity were significantly lower in disturbed stands compared to undisturbed ones (Fig. 3). However, PERMANOVA analyses showed significant differences in community composition between disturbed and undisturbed stands for bacteria (R^2^ = 0.08, F_1,28_ = 2.505, *p-value* = 0.002; Fig. 4A), fungi (R^2^ = 0.11, F_1,28_ = 3.293, *p-value* = 0.001; Fig. 4B) and microarthropod (R^2^ = 0.09, F_1,28_ = 1.991, *p-value* = 0.002; Fig. 4C). Small differences were observed in the composition of the dominant bacterial groups between disturbed and undisturbed stands. However, several fungal families (e.g., Russulaceae, Atheliaceae, Boletaceae, Elaphomycetaceae) and microarthropod families (e.g., Eupodidae, Tydeidae) were more abundant in undisturbed or disturbed stands (Fig. 5). To further explore changes in community composition, a differential abundance analysis was conducted. Six bacterial, five fungal and one microarthropod family were identified as bioindicators of disturbed stands, meaning they were discriminately more abundant in disturbed than undisturbed areas (Table 5). This analysis also highlighted that some dominant groups, notably the fungal family Elaphomycetaceae and the microarthropod family Tydeidae, showed a lower relative abundance in disturbed stands (Table 5; Fig. 5). Microbial and microarthropod alpha-diversity metrics and community composition did not change with time since infestation (Tables S7 and S8), meaning that soil biodiversity did not significantly change over time. Finally, disturbed and undisturbed areas differed in soil chemistry, with disturbed stands exhibiting higher C:N ratio, soil organic matter, total carbon and total nitrogen contents (Table S9).

**Table 5:** Discriminant ^a^bacterial, ^b^fungal and ^c^microarthropod families between undisturbed and disturbed stands in the Bavarian Forest National Park, according to Aldex2 differential abundance analysis. The mean relative abundance (%) (mean ± sd) of each discriminant family is indicated

Phylum	Class	Order	Family	Effect size	Undisturbed	Disturbed
^***a***^*Proteobacteria*	*Gammaproteobacteria*	*KF-JG30-C25*	*KF-JG30-C25*	1.23	0.06 ± 0.07	0.48 ± 0.30
^***a***^*Proteobacteria*	*Alphaproteobacteria*	*Rhodospirillales*	*Magnetospirillaceae*	1.17	0.04 ± 0.04	0.13 ± 0.09
^***a***^*Proteobacteria*	*Gammaproteobacteria*	*Burkholderiales*	*Gallionellaceae*	1.07	0.002 ± 0.004	0.130 ± 0.187
^***a***^*Verrucomicrobiota*	*Omnitrophia*	*Omnitrophales*	*Omnitrophaceae*	1.05	0.06 ± 0.03	0.22 ± 0.12
^***a***^*Fibrobacterota*	*Fibrobacteria*	*Fibrobacterales*	*Fibrobacteraceae*	1.06	0.05 ± 0.05	0.16 ± 0.11
^***a***^*Bacteroidota*	*Bacteroidia*	*Sphingobacteriales*	*env.OPS_17*	1.01	0.10 ± 0.10	0.24 ± 0.13
^***a***^*Patescibacteria*	*Parcubacteria*	*Candidatus_Jorgensenbacteria*	*Candidatus_Jorgensenbacteria*	0.98	0.08 ± 0.04	0.23 ± 0.10
^***a***^*Verrucomicrobiota*	*Omnitrophia*	*Omnitrophales*	*Omnitrophales*	0.98	0.05 ± 0.03	0.17 ± 0.08
^***a***^*Patescibacteria*	*Parcubacteria*	*Candidatus_Adlerbacteria*	*Candidatus_Adlerbacteria*	0.85	0.01 ± 0.01	0.04 ± 0.02
^***a***^*Actinobacteriota*	*Actinobacteria*	*Corynebacteriales*	*Mycobacteriaceae*	−0.95	0.92 ± 0.50	0.26 ± 0.22
^***a***^*Actinobacteriota*	*Actinobacteria*	*Streptomycetales*	*Streptomycetaceae*	−0.97	0.15 ± 0.10	0.06 ± 0.05
^***a***^*Proteobacteria*	*Gammaproteobacteria*	*Salinisphaerales*	*Solimonadaceae*	−1.03	0.08 ± 0.04	0.03 ± 0.03
^***a***^*Actinobacteriota*	*Actinobacteria*	*Catenulisporales*	*Actinospicaceae*	−1.11	0.52 ± 0.21	0.15 ± 0.09
^***a***^*Actinobacteriota*	*Actinobacteria*	*Corynebacteriales*	*Thermomonosporaceae*	−1.22	0.03 ± 0.03	0.001 ± 0.002
^***a***^*Actinobacteriota*	*Actinobacteria*	*Streptosporangiales*	*Nocardiaceae*	−1.53	0.07 ± 0.04	0.01 ± 0.01
^***b***^*Basidiomycota*	*Agaricomycetes*	*Boletales*	*Boletaceae*	−0.96	5.94 ± 4.65	1.86 ± 3.83
^***b***^*Basidiomycota*	*Agaricomycetes*	*Agaricales*	*Hygrophoraceae*	−1.19	1.55 ± 2.58	0.02 ± 0.07
^***b***^*Ascomycota*	*Dothideomycetes*	*Mytilinidales*	*Gloniaceae*	−1.24	3.77 ± 4.70	1.16 ± 2.70
^***b***^*Basidiomycota*	*Agaricomycetes*	*Agaricales*	*Amanitaceae*	−1.35	2.52 ± 2.91	1.35 ± 3.59
^***b***^*Ascomycota*	*Eurotiomycetes*	*Eurotiales*	*Elaphomycetaceae*	−1.48	5.39 ± 6.95	0.36 ± 1.37
^***c***^*Arthropoda*	*Arachnida*	*Trombidiformes*	*Tydeidae*	−1.46	5.50 ± 5.24	0.19 ± 0.32

## Discussion

### Soil biodiversity had a limited response to deadwood volume

European temperate forests are progressively shifting towards increased deadwood stocks (Bujoczek et al. 2021). Yet, it remains unclear how deadwood volume affects soil biodiversity in forest stands, such as microbial and microarthropod communities. To our knowledge, our study is among the first to explore the relationship between deadwood volume and soil biodiversity at the forest stand level, showing initial evidence of a marginal effect of ground-lying deadwood.

Contrary to expectations, variations in deadwood volume were limited to changes in community composition (beta diversity) but were not associated with changes in alpha diversity. Our results align with previous studies that also found changes in soil microbial and microarthropod community composition with deadwood addition without noticing changes in their diversity (Čuchta et al. 2019; Gonzalez-Polo et al. 2013; Ruppert et al. 2023; van der Wal et al. 2017). As deadwood accumulates and decays, the surrounding soil is enriched in nutrients (Lasota et al. 2018; Piaszczyk et al. 2021), resulting in local alterations in soil conditions. In our study, increased deadwood stocks were associated with increased soil organic matter, carbon and nitrogen contents, along with a weak but significant influence of these soil properties on microbial and microarthropod communities. Via these changes, deadwood may support richer and more functionally diverse microbial and microarthropod communities (Prescott and Grayston 2013). By creating new microhabitats and ecological niches, deadwood may promote species replacement across different locations, leading to variation in beta diversity. In other words, a higher deadwood stock may drive species turnover without necessarily affecting overall species richness. Furthermore, contrary to our first hypothesis, we did not identify any specific taxa associated with high deadwood volumes in our study, suggesting that other aspects of soil biodiversity may be affected instead. For example, the accumulation of recalcitrant and complex compounds from deadwood decay has been shown to favour microbial communities equipped with the necessary genes to access and metabolise these compounds (Minnich et al. 2021; Olchowik et al. 2019; Perreault et al. 2021).

Our results also show that both soil pH and the C:N ratio influence soil biodiversity. Soil pH acts as an environmental filter by modifying nutrient availability and inducing direct physiological stress, leading to more specialised and less diverse soil communities (Rousk et al. 2010; Rousseau et al. 2024b). The C:N ratio, ranging from 16.50 to 26.50 in our study, primarily affects bacterial communities. Higher values within this range can indicate increasing nitrogen limitation, which may constrain bacterial growth and favour more oligotrophic taxa, while lower values may promote fast-growing, copiotrophic bacteria (Fierer et al. 2007).

Additionally, the generally marginal response of the soil communities in our study might result from the scale at which soil microbial and microarthropod communities respond to local environmental conditions. Previous studies sampled soil directly underneath or near deadwood (e.g., Huhta et al. 2012; van der Wal et al. 2017), and at this local scale, directly linked shifts in microbial communities to changes in soil organic matter content as deadwood decays (Minnich et al. 2021; Olchowik et al. 2019). As here we aimed to explicitly test whether the total deadwood volume in a forest stand (i.e., 30 × 30 m forest plot) affected soil biodiversity at this scale, we chose to randomly collect forest bulk soil within these forests instead of sampling directly underneath any observed logs.

Although microbial and microarthropod communities are known to respond to highly local microhabitat conditions (Perreault et al. 2021; Wang et al. 2023), previous studies have shown that effects may extend to some meters around hotspots of deadwood. For instance, some microphytageous families (e.g., Opiidae) have been found to be more abundant near ground-lying deadwood compared to the surrounding soil habitat (Čuchta et al. 2019; Gonzalez-Polo et al. 2013). Furthermore, soil fungi also showed a response to deadwood addition up to 2 m (Olchowik et al 2019; Van der Wal et al 2017). Yet, such effects may decay at even larger scales (> 8 m from log; Minnich et al. 2021), limiting the relations observed at the level of our plots.

Furthermore, it must be noted that our results, based on DNA metabarcoding, only provide information on taxonomic diversity, without indicating real abundances, densities, or functional changes. Thus, if deadwood volume mainly affects the abundance or activity of species, rather than their presence/absence, this may explain the lack of variation in our dataset. For example, ground-lying deadwood has been shown to stimulate microbial activity, particularly carbon mineralisation (Błońska et al. 2023; Minnich et al. 2021).

### Distinct and less diverse soil biodiversity following bark beetle outbreaks

Following bark beetle outbreaks, the disturbed areas harbour distinct soil communities, especially fungal communities, and decreased fungal and microarthropod diversity. These disturbed areas differ greatly from undisturbed areas (i.e., areas without bark beetle outbreaks) due to changes in forest and plant community structure. These environmental changes following bark beetle infestation include increased herbaceous and shrub cover, large amounts of deadwood, and greater canopy openness, all of which contribute to altered soil conditions (Štursová et al. 2014) through increased light, mineralisation and vegetation growth (Gandhi et al. 2022).

The decline in fungal richness may be associated with a reduced relative abundance of ectomycorrhizal fungi. Ectomycorrhizal fungi are root symbionts depending on living tree hosts (Smith and Read 2016), and their diversity is closely linked to the amount and size of living trees (Birch et al. 2023; Sterkenburg et al. 2019). Our observations align with our sampling of post-bark beetle outbreak plots, where, living spruce trees are limited or absent. We also show a clear decrease in the relative abundance of several ectomycorrhizal taxa (e.g., Boletaceae, Elaphomycetaceae, Amanitaceae, Hygrophoraceae) in disturbed stands consistent with previous studies observing similar shifts in fungal communities following bark beetle outbreaks (Custer et al. 2020; Veselá et al. 2019). However, the decline in fungal richness in our study contradicts other studies (e.g., Custer et al. 2020; Doerfler et al. 2020), though it is consistent with the findings of Pec et al. (2017), who also showed a similar trend in fungal richness. Furthermore, despite the large amount of deadwood, which would favour saprotrophic fungi, the canopy opening exposes ground-lying deadwood, other woody debris and soil to desiccation (Beudert et al. 2014; Hilmers et al. 2018). This likely contributes to the loss of fungal richness, as moisture is critical for fungal colonisation and growth (Deepika and Kothamasi 2015; Nilsen et al. 1998).

Similarly, the shifts in microarthropod communities were likely driven by changes in forest structure. While we expected a positive response due to a large amount of ground-lying deadwood, which should increase habitat heterogeneity and food availability, we instead found a lower microarthropod diversity in disturbed areas. This result is consistent with studies reporting similar trends when comparing microarthropod diversity between closed and opened forest areas (Junggebauer et al. 2024; Liu et al. 2019; Nardi et al. 2023). The negative impact of canopy openness on microarthropods is further evidenced by the lower relative abundance of the Tydeidae family in disturbed stands, a known bioindicator of closed-canopy environments (Ames et al. 2023). Without canopy protection, altered microclimatic conditions, such as direct sunlight and increased soil temperature that can reach lethal levels for microarthropods, may contribute to this decline (Seastedt and Crossley 1981).

Unlike fungi and microarthropods, which primarily respond to changes in forest structure, bacteria are expected to adapt to variations in substrate availability following bark beetle outbreaks, particularly changes in carbon input (Gandhi et al. 2022). Of the six bacterial families found to be significantly less abundant in disturbed stands, five belonged to the Actinobacteria phylum. Actinobacteria are known to correlate with changes in carbon availability (Fierer et al. 2007) and tend to thrive in soils with high levels of labile carbon, such as that derived from root exudates (Goldfarb et al. 2011). Their decline reflects shifts in carbon availability post-disturbance. Carbon inputs from rhizodeposits decrease due to bark beetle-induced mortality (Mikkelson et al. 2016). At the same time, recalcitrant carbon compounds accumulate in the soil organic layer (Kosunen et al. 2020), such as cellulose and lignin, which are the main structural compounds of deadwood (Bani et al. 2018). Furthermore, a higher C:N ratio and increased soil organic matter content were observed in our disturbed stands, supporting the idea that changes in carbon input and availability can drive shifts in bacterial communities toward more oligotrophic taxa. For example, we identified one family from the order Sphingobacteriales that was more abundant in disturbed stands, consistent with findings from Mikkelson et al. (2016) and known to include oligotrophic taxa. This further supports the idea that changes in carbon input may be a key driver in shaping soil bacterial communities. However, the ecological functions of many of these bacterial families remain poorly understood, limiting our ability to interpret the impact of bark beetle outbreaks on carbon cycling in the Bavarian Forest National Park.

Bark beetle-affected areas share common features, such as large deadwood volumes and open canopies, but other factors, particularly the diversity of shrubs and herbs as stands regenerate, can vary significantly. Plant diversity affects not only soil microarthropod diversity (Bonari et al. 2016; Winter et al. 2015) but also microbial communities, as further alterations in, for example, carbon input, can occur with forest regeneration. Unfortunately, sampling multiple disturbed areas with different conditions was not possible during this study and generalisations to other locations based on these data should be made with care. Further research capturing a variety of geographic locations and the heterogeneity of stands regenerating from bark beetle infestation could improve our understanding and predictions of long-term soil biodiversity and functional changes following bark beetle outbreaks.

## Conclusion

Deadwood, identified as a remote-sensing biodiversity product (Skidmore et al 2021) and a component of the Essential Biodiversity Variable “Habitat Structure” (Pereira et al. 2013), promotes forest biodiversity by releasing nutrients and providing additional habitats and food resources. However, our study shows that increasing deadwood stock has a limited impact on forest soil diversity at the forest stand level, with the deadwood effect diminishing with distance. Furthermore, large-scale disturbances such as bark beetle outbreaks, which create large and sudden amounts of deadwood, change soil community composition and reduce biodiversity. Observing no correlations between time since infestation (3 to 25 years) and soil biodiversity, our study sheds light on potential drivers of soil biodiversity following bark beetle outbreaks. Understanding the long-term effects of these outbreaks on soil biodiversity is crucial for forest management, especially as more forested areas are expected to be affected in Europe.

## Supplementary Information

Below is the link to the electronic supplementary material.

Supplentary file 1 (XLSX 29.6 KB)

Supplentary file 2 (DOCX 17.1 MB)

## Data Availability

The data that supports the findings of this study are available in the supplementary material of this article.
