# Supplementary material for: Ground-lying deadwood volume promotes soil beta diversity but not alpha diversity in European temperate forests
Source: Plant Soil. 2025 Jun 18;515(1):579–96. doi: 10.1007/s11104-025-07600-6 (PMC12660469; doi:10.1007/s11104-025-07600-6)
Supplement: Supplementary file 2 — Supplentary file 2 (DOCX 17.1 MB) [file 11104_2025_7600_MOESM2_ESM.docx]

**Article title:** Ground-lying deadwood volume promotes soil beta diversity but not alpha diversity in European temperate forests

**Journal:** Plant and Soil

**Authors:** Mélody Rousseau*, G. Arjen de Groot, Andrew K. Skidmore, Andjin Siegenthaler, Ivo Laros, Marco Heurich, Devara P. Adiningrat, Elnaz Neinavaz

**Corresponding author:** Mélody Rousseau, Faculty of Geo-Information Science and Earth Observation, University of Twente, 7522 NH Enschede, The Netherlands. Email address: [melody.rousseau.85@gmail.com](mailto:melody.rousseau.85@gmail.com)

**
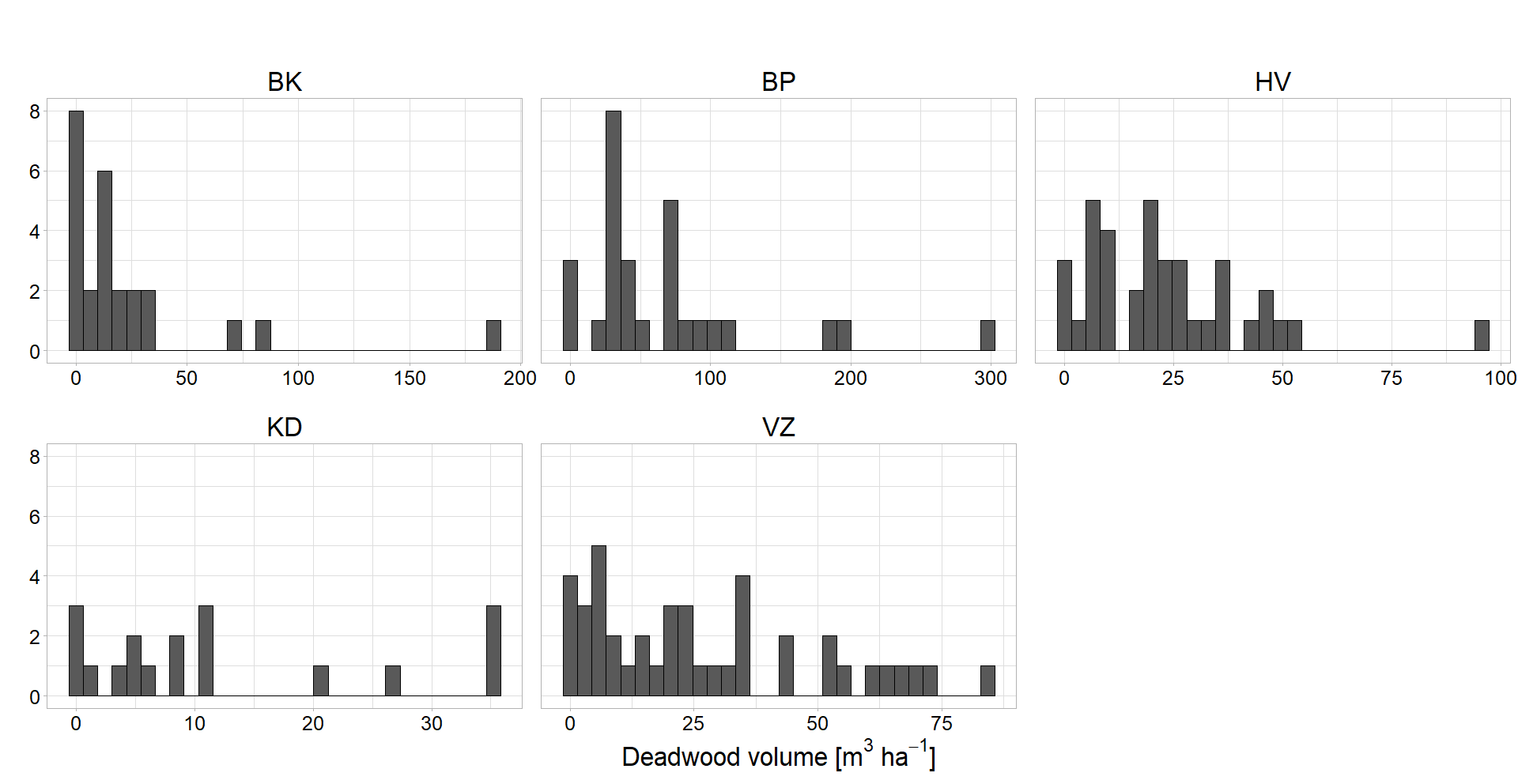
**

**Figure S1.** Histogram showing the deadwood volume (m^3^ ha^-1^) distribution for BK (Neuburg Forest), BP (Bavarian Forest National Park), HV (Hoge Veluwe National Park), KD (Royal Estate Het Loo) and VZ (Veluwezoom National Park).


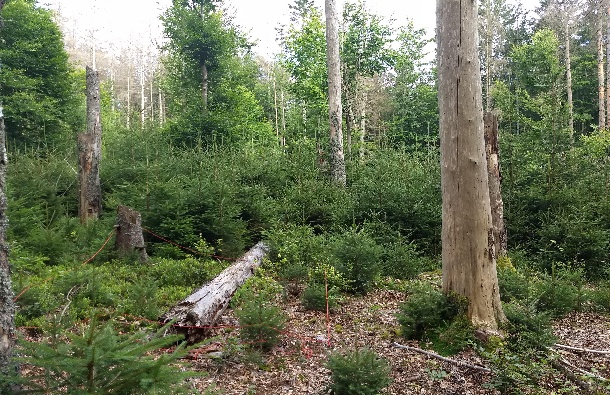

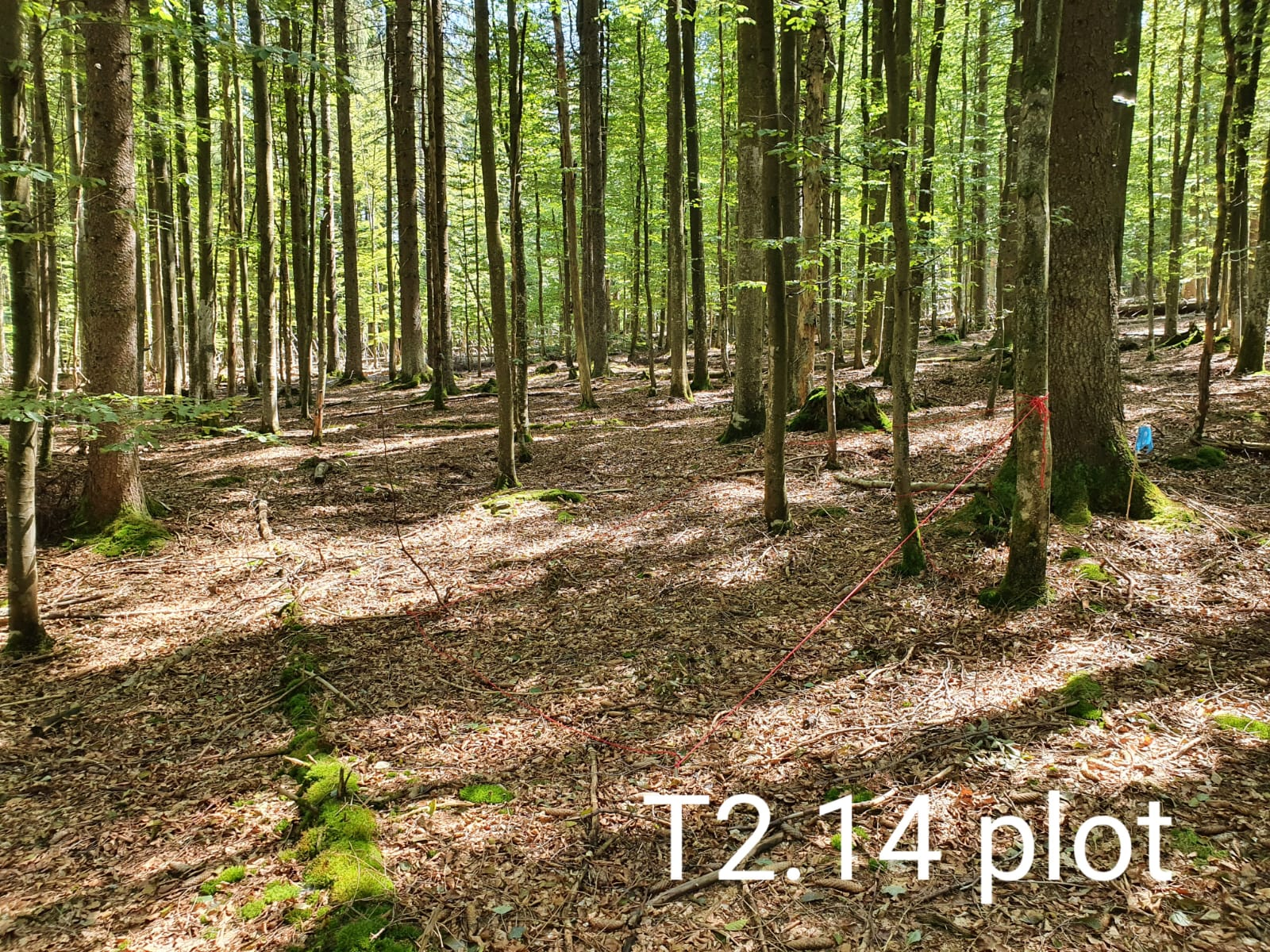

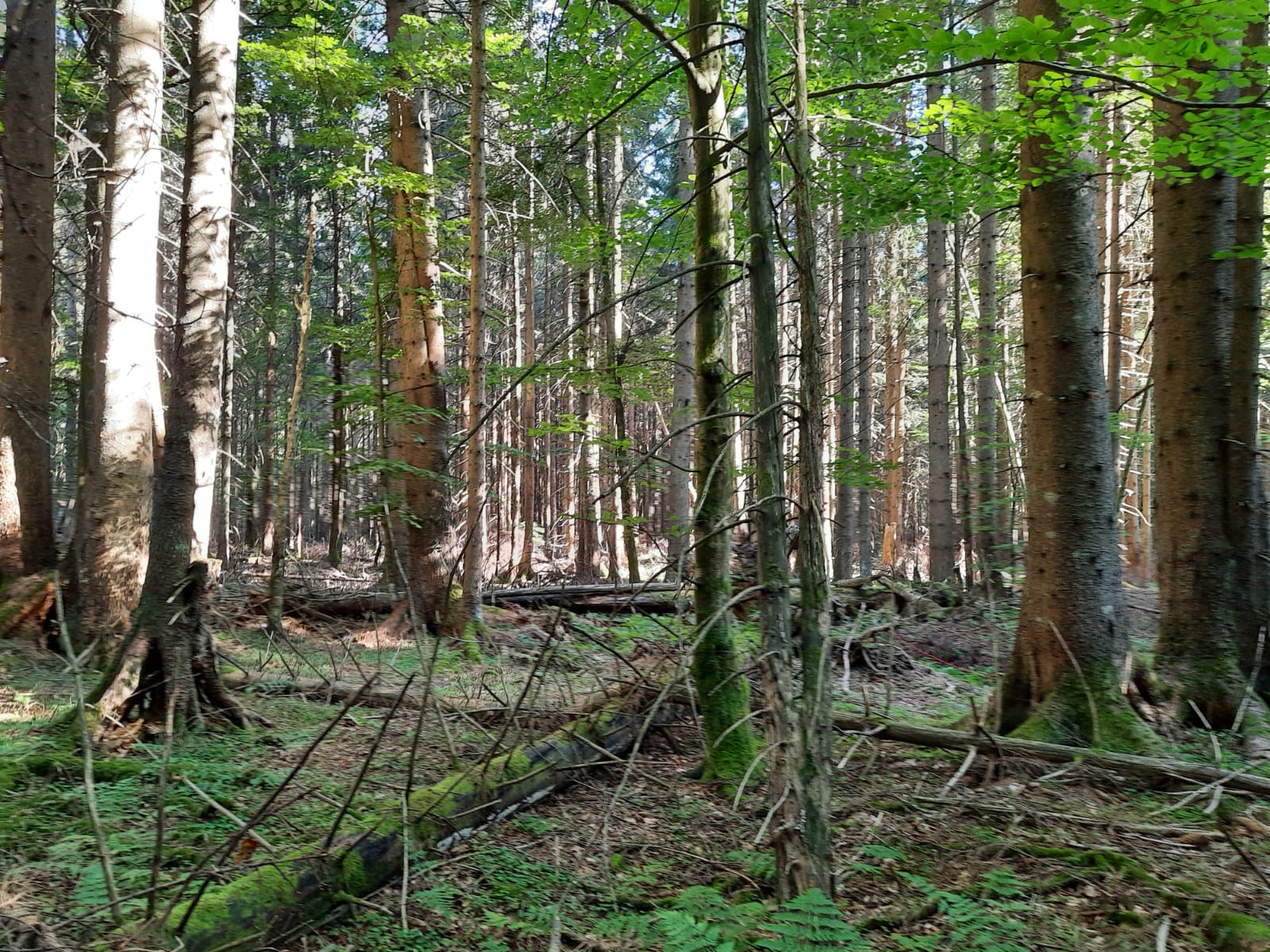

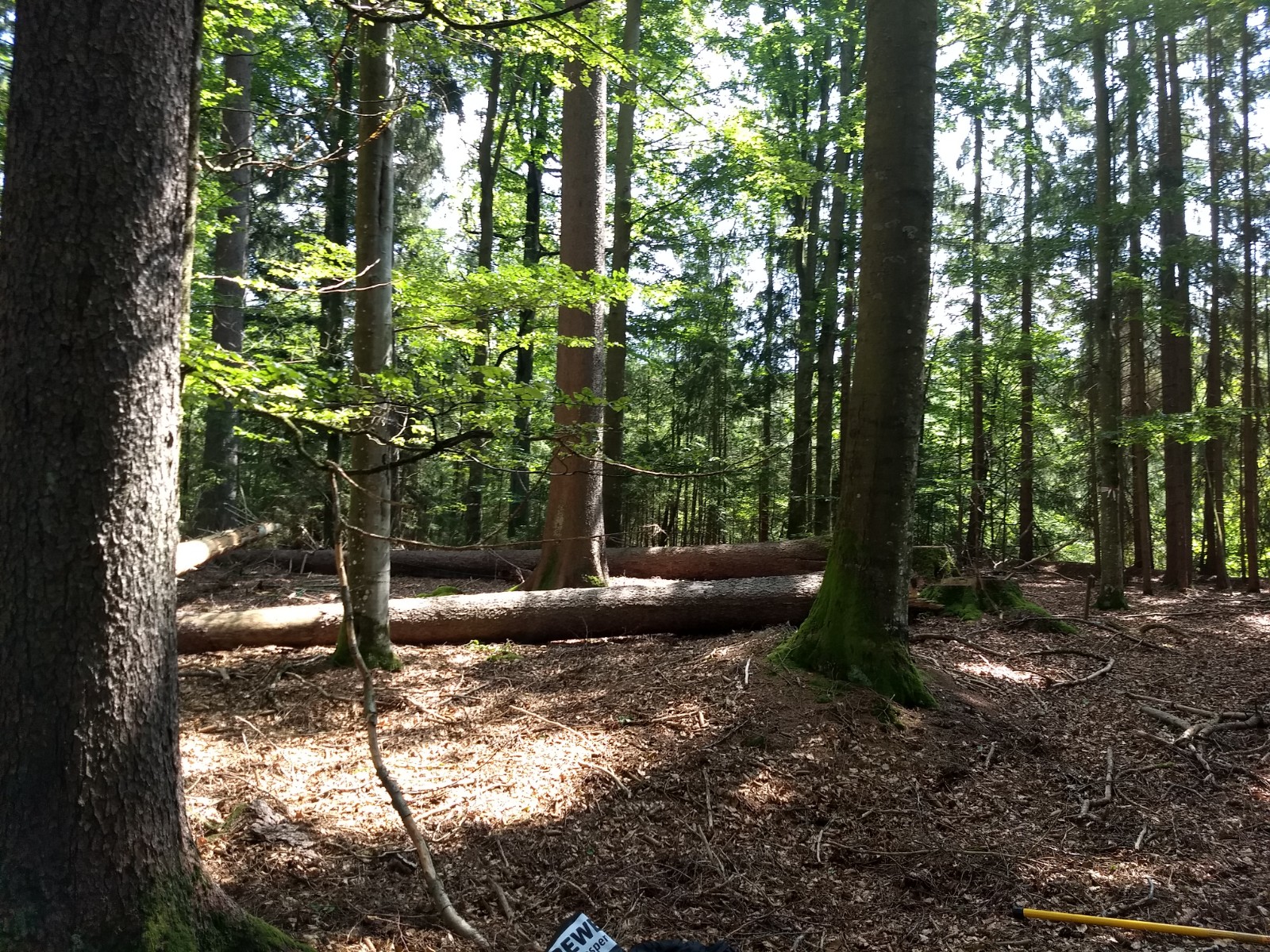


**Figure S2.** Pictures of four different sample plots showing the soil sampling design (subplot delimited by red ropes) and variation in deadwood volumes among plots.


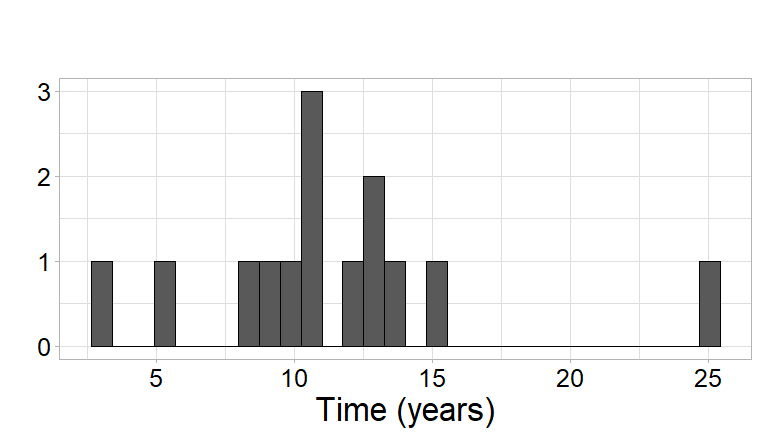


**Figure S3.** Histogram showing the time (years) since the bark beetle outbreaks for the 15 dieback Spruce plots in the Bavarian Forest National Park.


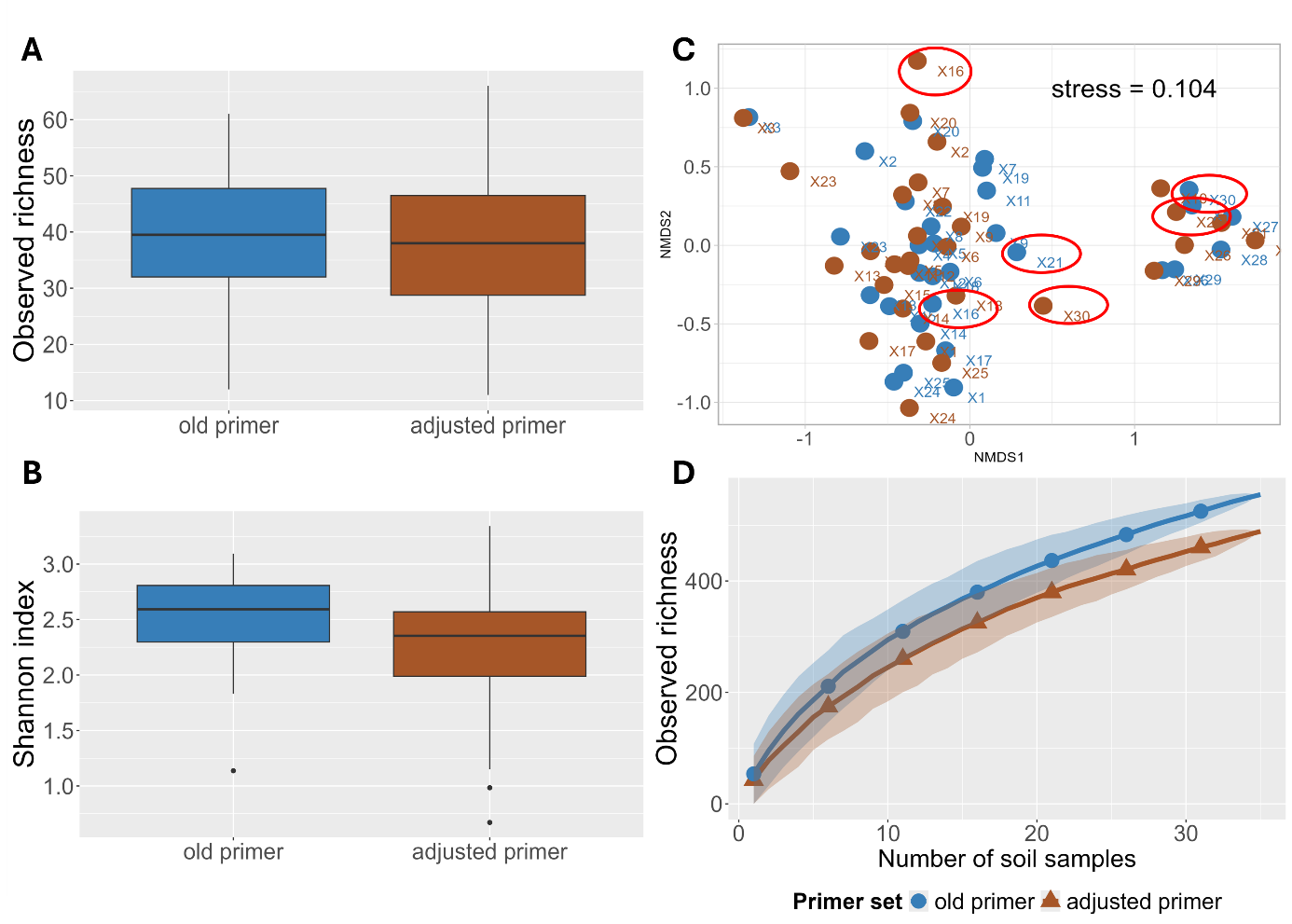
**Figure S4.** (A) Observed OTU richness and (B) Shannon index captured by the old and adjusted COI primer sets. (C) NMDS plot based on the Bray-Curtis dissimilarity at the OTU level (Hellinger-transformed reads) between the old and adjusted COI primer sets. The red circles highlight the three samples that showed considerable variation in community composition between primer sets. (D) OTU accumulation curves per primer set.


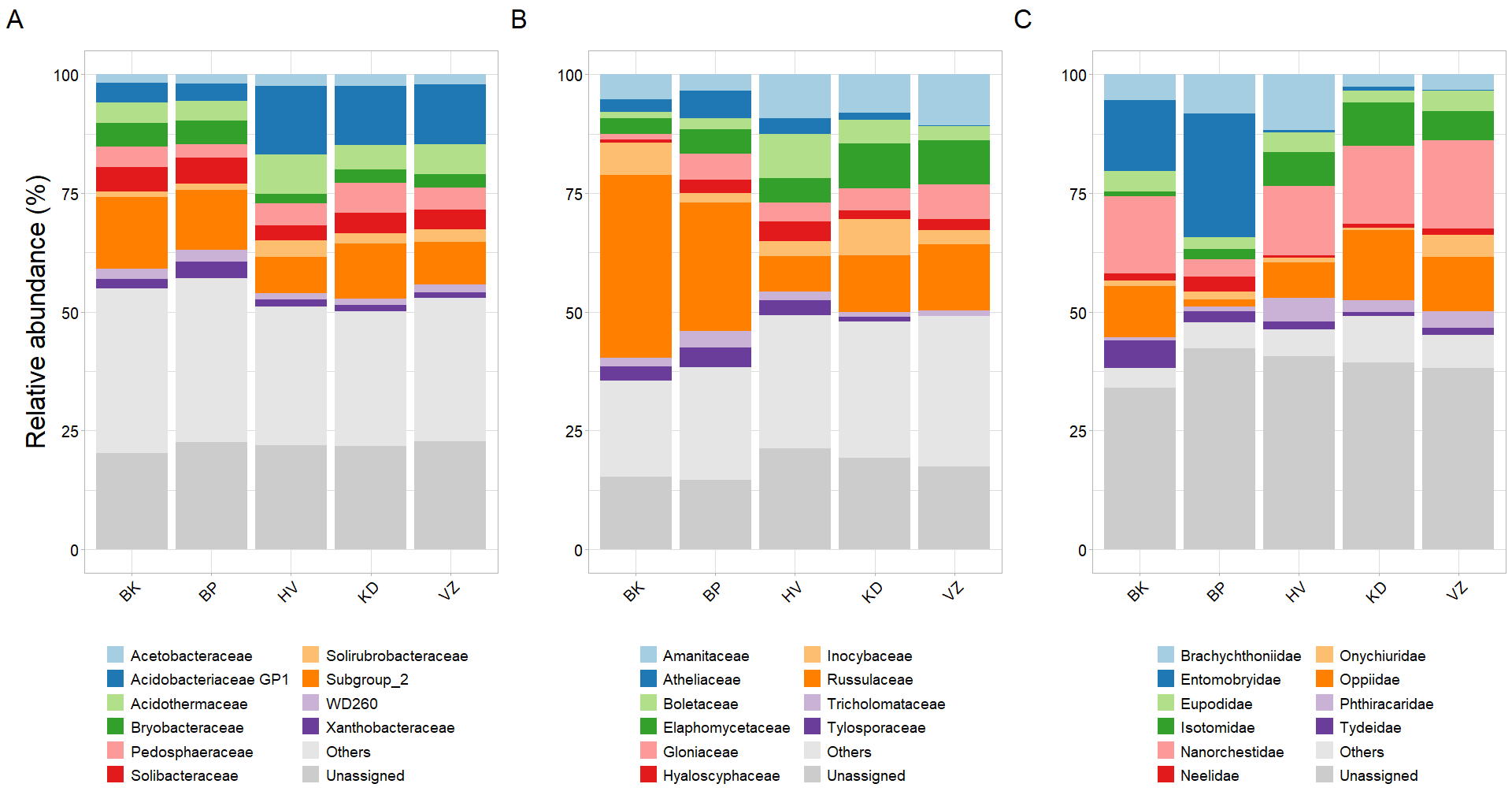


**Figure S5.** Mean relative abundance (%) of the top ten bacterial (A), fungal (B) and microarthropod (C) families between NF (Neuburg Forest), BP (Bavarian Forest National Park), HV (Hoge Veluwe National Park), KD (Royal Estate Het Loo) and VZ (Veluwezoom National Park).
